# Diagnostic accuracy of the WHO ICOPE step 1 screening tool in Korean community-dwelling older adults: a cross-sectional validation study

**DOI:** 10.3389/fpubh.2026.1897282

**Published:** 2026-07-30

**Authors:** HeeKyung Chang, MinJi Park, JuHee Seo, YoungJoo Do

**Affiliations:** College of Nursing, Gyeongsang National University, Jinju, Republic of Korea

**Keywords:** community-dwelling, diagnostic accuracy, healthy aging, ICOPE, intrinsic capacity, Korea, older adults, screening

## Abstract

**Background:**

The WHO ICOPE Step 1 tool screens decline in intrinsic capacity (IC), but its diagnostic accuracy has not been examined in Korea. This study evaluated four assessable non-sensory Step 1 domains against Korean-validated reference standards in Korean community-dwelling older adults.

**Methods:**

We conducted a cross-sectional study of 464 community-dwelling older adults (mean age 78.4 years; 67.9% female) from 11 community-based recruitment sites in urban and rural Jinju, South Korea. ICOPE Step 1 items were evaluated against reference standards in four assessable domains: cognition (Cognitive Impairment Screening Test [CIST], with age- and education-adjusted cutoffs), vitality (Mini-Nutritional Assessment Short-Form [MNA-SF] ≤ 11), psychological well-being (Geriatric Depression Scale [GDS-15] ≥ 8), and locomotion (Short Physical Performance Battery [SPPB] ≤ 9). Hearing and vision were reported descriptively because objective sensory reference standards were unavailable. Sensitivity, specificity, positive and negative predictive values, area under the curve (AUC), and Cohen’s kappa were calculated for each domain and for overall screening. Post-hoc analyses examined modified SPPB, BMI-augmented vitality screening, cognition calibration, adjusted urban–rural differences, recruitment-source sensitivity, and prevalence-adjusted predictive values.

**Results:**

Overall, Step 1 screening across four domains showed sensitivity of 89.0%, specificity of 45.1%, and AUC of 0.671 for any IC decline. In the original SPPB-based analysis, locomotion showed the highest apparent accuracy (sensitivity 70.0%, specificity 83.6%, AUC = 0.768, *κ* = 0.499), but modified SPPB analysis excluding the chair-stand component reduced AUC to 0.670, confirming incorporation bias. Psychological well-being showed AUC = 0.709, whereas cognition showed limited specificity (50.2%; low-education subgroup 45.7%). Adding BMI ≤ 20 kg/m^2^ to vitality screening increased sensitivity from 59.0 to 68.2% with minimal specificity loss. Adjusted analyses showed setting-dependent detection differences, especially lower locomotion detection in urban residents.

**Conclusion:**

Four assessable non-sensory ICOPE Step 1 domains showed acceptable overall case-finding sensitivity but heterogeneous domain-specific accuracy. Implementation should use education-stratified cognitive confirmation, BMI-augmented vitality screening, cautious interpretation of chair-rise locomotion results, and future objective validation of sensory domains.

## 1 Introduction

The World Health Organization (WHO) Integrated Care for Older People (ICOPE) framework marks a shift in geriatric care from disease-centered models toward a function-centered approach anchored in the concept of intrinsic capacity (IC) (1, 2). IC, defined as the composite of physical and mental capacities an individual can draw on at any point in life, comprises five core domains: cognition, locomotion, vitality, psychological well-being, and sensory function (hearing and vision) (3). The framework operationalizes IC assessment through a two-step pathway: Step 1 uses brief self-report and performance-based items to flag potential declines, and Step 2 applies standardized clinical instruments for confirmatory assessment (4).

Since its introduction in the 2017 ICOPE Guidelines, updated as a second edition in 2024 (5), Step 1 has been validated in several populations. Leung et al. (6) reported an overall sensitivity of 95% and a specificity of 57.6% in 304 community-dwelling older adults in Hong Kong. Rojano i Luque et al. (7) found variable domain-specific accuracy in 207 primary care patients in Spain: locomotion achieved the highest specificity (0.95), whereas vitality and sensory items performed poorly (sensitivity 0.44–0.56). Lu et al. (8) reported 94.6% sensitivity and 62.3% specificity in 228 Beijing residents aged ≥75 years. Most recently, Ma et al. (9) reported excellent overall performance (sensitivity 96.1%, specificity 92.3%) in nursing-home residents aged ≥80 years, though these figures partly reflect the very high prevalence of IC decline in institutional settings.

A critical gap remains, however, in the Korean context. Korea became a super-aged society in 2025, with more than 20% of its population aged ≥65 years (10). Korean geriatric research has yielded substantial IC-related work through the Korean Frailty and Aging Cohort Study (KFACS) and the ICOOP-Frail program (11, 12), and a recent Delphi study identified primary-care frailty and IC screening as the top priority for adapting ICOPE to the Korean health system (13). To date, no published study has evaluated the diagnostic accuracy of ICOPE Step 1 against comprehensive reference standards in a Korean community-dwelling population.

This gap matters because several factors may shape ICOPE Step 1 performance in the Korean context. Brief cognitive screening tasks that include word recall may function differently in populations with a high proportion of older adults with limited formal education (14). Korea’s urban–rural health disparities—driven by differential access to health services and distinct socioeconomic profiles—may also produce setting-dependent variation in screening accuracy (15). In addition, Korea has developed its own national cognitive screening instrument, the Cognitive Impairment Screening Test (CIST), with age- and education-adjusted cutoffs calibrated for the Korean population (16); the CIST therefore provides a more contextually appropriate reference standard than the Mini-Mental State Examination commonly used internationally.

We therefore evaluated the diagnostic accuracy of four assessable non-sensory domains of the WHO ICOPE Step 1 tool against Korean-validated reference standards in community-dwelling older adults from an urban–rural mixed region. Hearing and vision were examined descriptively because objective sensory reference standards were not available. Our specific aims were to (1) determine the sensitivity, specificity, predictive values, and agreement for each assessable non-sensory IC domain; (2) quantify overall screening accuracy for detecting any decline across these four domains; and (3) examine variation in diagnostic performance by sex, education level, residential area, and recruitment source.

## 2 Methods

### 2.1 Study design and setting

This cross-sectional diagnostic accuracy study was conducted in Jinju, a city in Gyeongnam Province, South Korea. Jinju is an urban–rural mixed municipality (DoNong Bokhap) that includes both urban districts (Eup/Dong) and rural districts (Myeon), and thus provides a pragmatic urban–rural mixed setting for evaluating ICOPE screening across common Korean community service environments. Data were collected between October 2025 and January 2026 as part of the Digital Rural–urban Ecosystem for Active Management and Successful Aging (DREAMS) project, a multi-year community-based program on healthy aging funded by the National Research Foundation of Korea.

### 2.2 Participants

We recruited 464 community-dwelling older adults from 11 community-based recruitment sites grouped into four functional categories: two senior welfare centers (*n* = 81), one primary-care outpatient clinic (*n* = 157), three older-adult day-care centers (*n* = 122), and five senior community halls or village centers (*n* = 104). No participants were recruited from rehabilitation hospitals. All participants lived at home in the community and were ambulatory at the time of recruitment. Senior welfare centers and community halls served as congregate activity sites for independent older adults. Day-care centers in Korea operate as community social-welfare facilities providing daytime supervision and health-promotion activities for older adults who live at home. The primary-care outpatient clinic contributed registered outpatients from surrounding neighborhoods. Data collection was conducted by the same trained research team using identical protocols across all locations. Eligible participants were aged ≥65 years, lived in Jinju, and were able to give informed consent and complete the survey instruments. We excluded individuals with acute illness at assessment, severe cognitive impairment precluding informed consent, or current hospitalization. Purposive sampling was used to include both urban (*n* = 268, 57.8%) and rural (*n* = 196, 42.2%) residents.

### 2.3 Ethical considerations

The study was approved by the Institutional Review Board of Gyeongsang National University (IRB No. GIRB-G25-NW-0112) and conducted in accordance with the Declaration of Helsinki. All participants provided written informed consent before data collection.

### 2.4 ICOPE step 1 screening (index test)

The WHO ICOPE Step 1 tool was administered according to the standardized protocol in the ICOPE handbook (4). It assesses six conditions across five IC domains through brief questions and performance-based tasks. (1) Cognition was screened with a three-word recall task (the words flower, door, and rice). (2) Vitality was assessed with two items: self-reported unintentional weight loss of ≥3 kg in the past 3 months, and self-reported poor appetite. (3) Psychological well-being was assessed with two items: feeling sad, down, or depressed during the past 2 weeks, and loss of interest or pleasure in previously enjoyed activities. (4) Hearing and (5) vision were screened by asking whether the participant had any difficulty hearing or seeing, respectively. (6) Locomotion was assessed with the chair-rise test (five repetitions from a seated position). Each domain was coded as screen-positive (potential decline) or screen-negative (no apparent decline); for vitality and psychological well-being, a positive response on either component item was sufficient to classify the domain as positive.

### 2.5 Reference standards (step 2 equivalent assessments)

Four IC domains were evaluated against comprehensive, Korean-validated reference standards. The sensory domains (hearing and vision) were excluded from the diagnostic accuracy analysis because objective audiometric and ophthalmologic assessments were not performed; results for these domains are reported descriptively only.

*Cognition*. The Cognitive Impairment Screening Test (CIST) (16), developed by the Korean Ministry of Health and Welfare with the National Institute of Dementia, served as the reference standard. The CIST provides age- and education-adjusted normative cutoffs for suspected cognitive impairment. For participants with low educational attainment (≤elementary-school completion), the cutoffs were 22, 20, and 18 for age groups <70, 70–79, and ≥80 years, respectively. For participants with higher educational attainment (≥middle school), the cutoffs were 24, 22, and 20 for the same age groups. Scores at or below the relevant cutoff were classified as cognitive impairment.

*Vitality/Nutrition*. The Mini-Nutritional Assessment Short-Form (MNA-SF) (17) served as the reference standard for the vitality domain. MNA-SF scores range from 0 to 14, with scores ≤11 indicating at-risk or malnourished status; a score ≤11 was therefore used to define vitality decline.

*Psychological well-being*. The 15-item Geriatric Depression Scale (GDS-15) (18) served as the reference standard, with a cutoff of ≥8 used to define clinically significant depressive symptoms, consistent with the validated Korean threshold for community screening (19).

*Locomotion*. The Short Physical Performance Battery (SPPB) (20) served as the reference standard. The SPPB assesses balance, gait speed, and chair-stand performance on a 0–12 scale; a score ≤9 was used to define locomotor impairment, corresponding to moderate-to-severe lower-extremity functional limitation. Of note, the SPPB includes a chair-stand component (scored 0–4) that structurally overlaps with the ICOPE Step 1 locomotion item (IC6). A post-hoc sensitivity analysis using a modified SPPB score excluding the chair-stand component (balance + gait speed only, range 0–8, cutoff ≤6) was conducted to quantify the resulting bias (Section 3.6; Supplementary Table S4).

### 2.6 Other measures

Sociodemographic data included age, sex, educational attainment (no formal education, elementary school, middle school, high school, or college/above), and residential area (urban or rural district). Anthropometric measures included body weight, height, and body mass index (BMI). Grip strength was measured with a hand dynamometer, and the mean of bilateral measurements was recorded. The Timed Up and Go test (TUG) (21) and the Five-Times Sit-to-Stand Test (FTSST) were administered as supplementary physical-performance measures, and the Simplified Nutritional Appetite Questionnaire (SNAQ) (22) was used to assess appetite. Self-reported medical histories of diagnosed hearing loss and cataract/glaucoma were collected using the chronic disease checklist. These variables were not based on chart review, audiometry, or ophthalmologic examination.

### 2.7 Statistical analysis

Diagnostic accuracy was calculated for each of the four assessable IC domains and for overall screening, defined as positivity on any of these four domains. Sensitivity, specificity, PPV, NPV, accuracy, Youden index, likelihood ratios, AUC, and Cohen’s *κ* were derived from 2 × 2 contingency tables. Cohen’s κ was interpreted as slight (0.00–0.20), fair (0.21–0.40), moderate (0.41–0.60), substantial (0.61–0.80), or almost perfect (0.81–1.00) (25). Ninety-five percent confidence intervals were estimated using 2,000 bias-corrected bootstrap resamples. Subgroup estimates were calculated by sex, education level, and residential area and were interpreted descriptively. Group differences in participant characteristics were tested using independent-samples t-tests for continuous variables and chi-square tests for categorical variables.

Post-hoc analyses addressed reviewer-identified methodological concerns. First, a modified SPPB sensitivity analysis excluded the chair-stand component from the locomotion reference standard. Second, BMI-augmented vitality screens were evaluated and compared with the standard vitality screen using McNemar-equivalent paired tests. Third, cognition screening was compared against both education-adjusted CIST cutoffs and an unadjusted raw CIST cutoff, and logistic regression evaluated whether the ICOPE cognition item retained diagnostic value after adjustment for education. Fourth, urban–rural differences in detection among reference-positive participants were evaluated using multivariable logistic regression with residential area as the primary predictor and age, education level, and sex as covariates. Fifth, prevalence-adjusted PPV and NPV were calculated across hypothetical community prevalence scenarios using Bayes’ theorem. Sixth, recruitment-source sensitivity analyses excluded the primary-care outpatient site and used Firth penalized logistic regression with Benjamini–Hochberg false-discovery-rate correction.

All analyses were performed in R 4.5.1. This study was reported in accordance with the STARD 2015 guidelines (Supplementary Table S5).

## 3 Results

### 3.1 Participant characteristics

A total of 464 community-dwelling older adults participated. The mean age was 78.4 years (SD = 7.6; range 65–105), and 315 (67.9%) were female. Nearly three-quarters of participants (*n* = 338, 72.8%) had low educational attainment (≤elementary-school completion). Urban residents accounted for 57.8% (*n* = 268) of the sample and rural residents for 42.2% (*n* = 196). Compared with urban participants, rural participants were significantly older (80.2 vs. 77.0 years, *p* < 0.001), had lower educational attainment (78.6% vs. 68.6% with ≤elementary schooling, *p* = 0.023), and had lower CIST scores (18.8 vs. 21.8, *p* < 0.001), SPPB scores (7.7 vs. 8.9, *p* < 0.001), and grip strength (17.9 vs. 21.9 kg, *p* < 0.001). In contrast, rural participants reported lower GDS-15 scores (4.8 vs. 6.3, *p* < 0.001) and higher SNAQ scores (14.3 vs. 13.4, *p* < 0.001). Full participant characteristics are shown in Table 1. Participant characteristics by recruitment-site category are presented in Supplementary Table S6.

**Table 1:** Participant characteristics (*N* = 464).

Characteristic	Total (*N* = 464)	Urban (*n* = 268)	Rural (*n* = 196)	*p*
Age, years, mean (SD)	78.4 (7.6)	77.0 (6.1)	80.2 (8.9)	<0.001
65–74, *n* (%)	149 (32.1)	90 (33.6)	59 (30.1)	
75–84, *n* (%)	213 (45.9)	147 (54.9)	66 (33.7)	
≥85, *n* (%)	102 (22.0)	31 (11.6)	71 (36.2)	<0.001
Female, *n* (%)	315 (67.9)	172 (64.2)	143 (73.0)	0.057
Education, *n* (%)				
No formal education	195 (42.0)	92 (34.3)	103 (52.6)	
Elementary school	143 (30.8)	92 (34.3)	51 (26.0)	
≥Middle school	126 (27.2)	84 (31.3)	42 (21.4)	0.023
BMI, kg/m^2^, mean (SD)	24.1 (3.3)	24.1 (3.0)	24.0 (3.5)	0.791
Grip strength, kg, mean (SD)	20.2 (9.2)	21.9 (8.6)	17.9 (9.5)	<0.001
Hypertension, *n* (%)	264 (56.9)	154 (57.5)	110 (56.1)	0.781
Diabetes, *n* (%)	128 (27.6)	83 (31.0)	45 (23.0)	0.057
Assessment scores, mean (SD)				
CIST total	20.6 (7.2)	21.8 (7.2)	18.8 (6.8)	<0.001
MNA-SF total	11.2 (2.5)	11.0 (2.7)	11.4 (2.2)	0.042
GDS-15 total	5.7 (2.8)	6.3 (2.9)	4.8 (2.5)	<0.001
SPPB total	8.4 (2.5)	8.9 (2.5)	7.7 (2.2)	<0.001
SNAQ total	13.8 (2.8)	13.4 (3.0)	14.3 (2.2)	<0.001
TUG, seconds	13.4 (5.3)	12.1 (4.2)	15.0 (6.2)	<0.001

BMI, body mass index; CIST, cognitive impairment screening test; MNA-SF, mini-nutritional assessment short-form; GDS-15, geriatric depression scale 15-item; SPPB, short physical performance battery; SNAQ, simplified nutritional appetite questionnaire; TUG, timed up and go test. *p*-values from independent-samples t-tests (continuous) or chi-square tests (categorical). Age group and education *p*-values refer to overall distribution differences.

### 3.2 Prevalence of IC decline by step 1 screening and reference standards

Table 2 shows the prevalence of IC decline as identified by Step 1 screening and by the reference standards. Overall, 83.0% of participants screened positive on at least one Step 1 domain, compared with 82.3% classified as having decline on at least one reference standard. Locomotion showed the highest reference-defined prevalence (63.1%), followed by vitality (46.8%), cognition (41.2%), and psychological well-being (25.6%). Step 1 tended to over-detect cognitive decline (59.3% vs. 41.2% by reference) and psychological decline (41.2% vs. 25.6%), and to under-detect locomotor decline (50.2% vs. 63.1%). For the self-reported sensory items, 45.0% of participants reported hearing difficulty and 64.7% reported vision difficulty. These proportions exceeded the prevalence of self-reported medical diagnoses of hearing loss (6.2%) and cataract/glaucoma (12.9%) captured through the chronic disease checklist. This comparison represents perceived current difficulty versus recalled diagnostic history, both based on self-report, rather than validation against objective clinical assessment; the full cross-tabulation is provided in Supplementary Table S2, and the distribution of the number of domains with detected decline is shown in Supplementary Table S3.

**Table 2:** Prevalence of intrinsic capacity decline by ICOPE step 1 screening and reference standard assessments.

Domain	Step 1 positive, *n* (%)	Reference positive, *n* (%)	Reference standard
Cognition	275 (59.3)	191 (41.2)	CIST (age/edu-adjusted)
Vitality	202 (43.5)	217 (46.8)	MNA-SF ≤ 11
Psychological	191 (41.2)	119 (25.6)	GDS-15 ≥ 8
Locomotion	233 (50.2)	293 (63.1)	SPPB ≤9
Any (4 domains)	385 (83.0)	382 (82.3)	Any reference positive
Hearing*	209 (45.0)	29 (6.2)^†^	Self-reported medical diagnosis
Vision*	300 (64.7)	60 (12.9)^‡^	Self-reported medical diagnosis

*Sensory domains reported descriptively only; no objective audiometric or ophthalmologic reference standard was available. ^†^Self-reported medical history of diagnosed hearing loss. ^‡^Self-reported medical history of diagnosed cataract/glaucoma.

### 3.3 Diagnostic accuracy of ICOPE step 1 screening

Table 3 shows the diagnostic accuracy metrics for each domain and for overall screening, and the corresponding 2 × 2 contingency tables are provided in Supplementary Table S1. In the original SPPB-based analysis, locomotion showed the highest apparent diagnostic performance, with a sensitivity of 70.0%, specificity of 83.6%, PPV of 88.0%, and AUC of 0.768. Agreement between the Step 1 chair-rise test and the SPPB reference was moderate (*κ* = 0.499). Because the SPPB includes a chair-stand component that overlaps with the ICOPE Step 1 chair-rise item, these primary estimates should be interpreted together with the modified SPPB sensitivity analysis reported in Section 3.6.

**Table 3:** Diagnostic accuracy of ICOPE step 1 screening against reference standards (*N* = 464).

Domain	Sn	Sp	PPV	NPV	Acc	Youden	LR+	LR–	AUC	*κ*
Cognition	0.728 [0.66, 0.79]	0.502 [0.44, 0.56]	0.505 [0.45, 0.56]	0.725 [0.66, 0.79]	0.595 [0.55, 0.64]	0.230 [0.14, 0.32]	1.46 [1.26, 1.69]	0.54 [0.41, 0.69]	0.615 [0.57, 0.66]	0.215 [0.13, 0.30]
Vitality	0.590 [0.53, 0.66]	0.700 [0.64, 0.76]	0.634 [0.57, 0.70]	0.660 [0.60, 0.72]	0.649 [0.60, 0.69]	0.290 [0.21, 0.38]	1.97 [1.59, 2.50]	0.59 [0.48, 0.69]	0.645 [0.60, 0.69]	0.291 [0.21, 0.38]
Psychological	0.723 [0.64, 0.81]	0.696 [0.64, 0.74]	0.450 [0.38, 0.52]	0.879 [0.84, 0.92]	0.703 [0.66, 0.74]	0.418 [0.32, 0.51]	2.37 [1.97, 2.91]	0.40 [0.28, 0.52]	0.709 [0.66, 0.76]	0.349 [0.27, 0.44]
Locomotion	0.700 [0.65, 0.75]	0.836 [0.77, 0.89]	0.880 [0.83, 0.92]	0.619 [0.56, 0.68]	0.750 [0.71, 0.79]	0.536 [0.46, 0.61]	4.27 [3.05, 6.16]	0.36 [0.29, 0.43]	0.768 [0.73, 0.81]	0.499 [0.42, 0.58]
Any domain	0.890 [0.86, 0.92]	0.451 [0.34, 0.56]	0.883 [0.85, 0.91]	0.468 [0.36, 0.58]	0.812 [0.77, 0.85]	0.341 [0.23, 0.45]	1.62 [1.35, 2.03]	0.24 [0.17, 0.36]	0.671 [0.62, 0.73]	0.346 [0.24, 0.45]

Sn, sensitivity; Sp, specificity; PPV, positive predictive value; NPV, negative predictive value; Acc, accuracy; LR+, positive likelihood ratio; LR–, negative likelihood ratio; AUC, area under the receiver operating characteristic curve; *κ*, Cohen’s kappa.

The psychological domain ranked second, with a sensitivity of 72.3%, a specificity of 69.6%, and an AUC of 0.709. Agreement was only fair (*κ* = 0.349), but the high NPV of 87.9% indicates that a negative screen on the depression/anhedonia items reliably rules out GDS-defined depressive symptoms.

The cognition domain performed worst, with a sensitivity of 72.8% but a specificity of only 50.2% and an AUC of 0.615. Agreement was slight-to-fair (*κ* = 0.215), and the low specificity reflected a high false-positive rate: 136 of 273 participants without CIST-defined cognitive impairment (49.8%) screened positive on the three-word recall item.

The vitality domain showed moderate accuracy, with a sensitivity of 59.0%, a specificity of 70.0%, and an AUC of 0.645; the combined weight-loss and appetite items captured just under 60% of participants with MNA-SF-defined nutritional risk.

**Figure 1:**
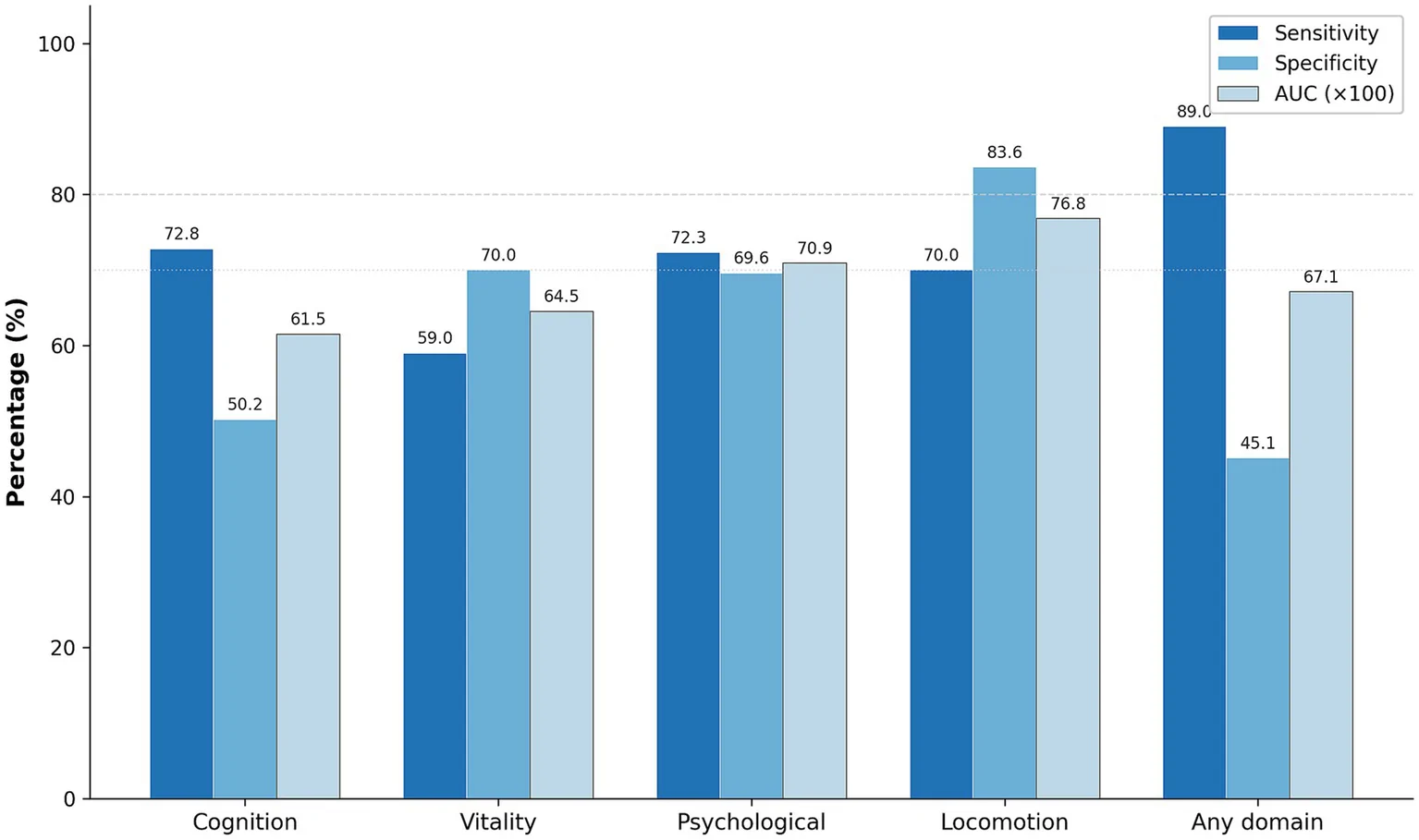
Diagnostic performance of ICOPE Step 1 screening across four intrinsic capacity domains and overall. Sensitivity, specificity, and area under the receiver operating characteristic curve (AUC, scaled ×100 for visual comparability) are shown for each domain. Dashed horizontal reference lines at 70% and 80% are provided for visual reference. Locomotion values are based on the original SPPB reference standard and should be interpreted together with the modified SPPB sensitivity analysis excluding the chair-stand component.

Across the four assessable domains, ICOPE Step 1 reached a sensitivity of 89.0% for detecting any domain-level IC decline, with a specificity of 45.1% and an AUC of 0.671. Figure 1 summarizes the domain-level diagnostic performance metrics.

### 3.4 Subgroup analyses

Table 4 presents the sensitivity and specificity of ICOPE Step 1 screening across subgroups defined by education, sex, and residential area.

**Table 4:** Sensitivity and specificity of ICOPE step 1 screening by subgroup.

Subgroup	Cognition		Vitality		Psychological		Locomotion	
	Sn	Sp	Sn	Sp	Sn	Sp	Sn	Sp
Education								
Low (≤Elem, *n* = 338)	0.761 [0.69, 0.83]	0.457 [0.38, 0.54]	—	—	—	—	—	—
High (≥middle, *n* = 126)	0.536 [0.36, 0.72]	0.582 [0.48, 0.68]	—	—	—	—	—	—
Sex								
Female (*n* = 315)	0.772 [0.70, 0.84]	0.441 [0.37, 0.52]	0.620 [0.54, 0.69]	0.645 [0.56, 0.72]	0.701 [0.60, 0.79]	0.675 [0.61, 0.74]	0.726 [0.67, 0.78]	0.837 [0.75, 0.91]
Male (*n* = 149)	0.618 [0.48, 0.74]	0.617 [0.52, 0.72]	0.500 [0.36, 0.62]	0.789 [0.70, 0.87]	0.781 [0.62, 0.90]	0.735 [0.65, 0.81]	0.614 [0.50, 0.73]	0.835 [0.75, 0.92]
Residential area								
Urban (*n* = 268)	0.830 [0.74, 0.90]	0.528 [0.45, 0.60]	0.679 [0.59, 0.76]	0.657 [0.57, 0.74]	0.700 [0.60, 0.79]	0.680 [0.61, 0.75]	0.532 [0.45, 0.61]	0.882 [0.82, 0.93]
Rural (*n* = 196)	0.641 [0.55, 0.74]	0.452 [0.35, 0.56]	0.446 [0.34, 0.56]	0.752 [0.66, 0.83]	0.793 [0.63, 0.93]	0.713 [0.64, 0.78]	0.855 [0.80, 0.91]	0.705 [0.56, 0.83]

Sn, sensitivity; Sp, specificity. Education-stratified analysis shown for cognition only. —, not separately computed.

*Education-stratified cognition performance*. Among participants with low education (≤elementary school; *n* = 338), cognition sensitivity was 76.1%, but specificity fell to 45.7%. Among those with higher education (≥middle school; *n* = 126), sensitivity was lower at 53.6%, while specificity improved slightly to 58.2%.

*Sex differences*. Female participants had higher cognition sensitivity than male participants (77.2% vs. 61.8%) but lower specificity (44.1% vs. 61.7%). For psychological screening, sensitivity was higher in men (78.1% vs. 70.1%). Locomotion sensitivity was higher in women (72.6% vs. 61.4%), with comparable specificity in both sexes.

**Figure 2:**
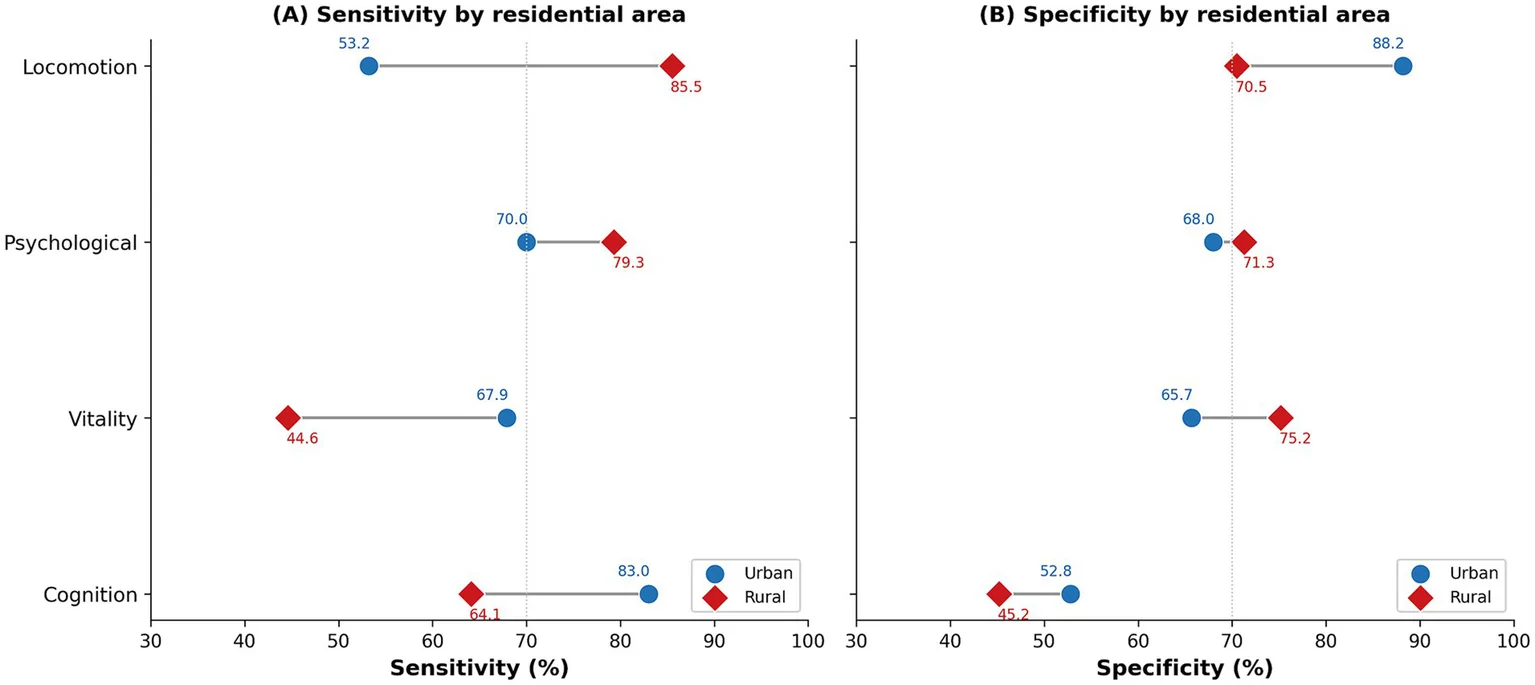
Urban–rural comparison of ICOPE Step 1 diagnostic performance. **(A)** Sensitivity and **(B)** specificity for each domain are displayed as paired dot plots (dumbbell charts). Blue circles represent urban residents (*n* = 268); red diamonds represent rural residents (*n* = 196). Connected lines show unadjusted urban–rural differences; adjusted urban–rural detection models are reported in Table 5. A dotted vertical reference line at 70% is provided for visual reference.

*Urban–rural differences*. In unadjusted subgroup estimates, cognition sensitivity was higher in urban than in rural residents (83.0% vs. 64.1%), whereas locomotion sensitivity was higher in rural than in urban residents (85.5% vs. 53.2%). Vitality sensitivity was also higher in urban than in rural residents (67.9% vs. 44.6%), whereas psychological screening was comparable between settings. Figure 2 displays the unadjusted urban–rural sensitivity and specificity differences across the four domains; adjusted urban–rural models are reported in Section 3.7 and Table 5.

**Table 5:** Adjusted urban–rural differences in detection among reference-positive participants.

Domain	Ref+ N	Urban Sn	Rural Sn	Adjusted OR [95% CI]	*p*
Cognition	191	83.0%	64.1%	4.821 [2.239, 11.154]	<0.001
Vitality	217	67.9%	44.6%	4.096 [2.160, 8.031]	<0.001
Psychological	119	70.0%	79.3%	0.687 [0.226, 1.870]	0.480
Locomotion	293	53.2%	85.5%	0.211 [0.111, 0.387]	<0.001

Logistic regression: detected ~ urban + age + education + female. OR>1 = higher detection in urban. VIFs <1.54.

### 3.5 Cognition domain: education-adjusted sensitivity analysis

Comparing IC1 against unadjusted raw CIST scores (fixed cutoff <20 points) yielded virtually identical diagnostic accuracy: AUC = 0.613 (raw) versus 0.615 (education-adjusted), with all difference confidence intervals crossing zero (Table 6). This indicates that the low cognition specificity reflects an inherent limitation of the 3-word recall task rather than a statistical artifact. Logistic regression with CIST-defined cognitive impairment as the outcome confirmed that IC1 retained independent diagnostic value after education adjustment (OR = 2.219, 95% CI 1.471–3.371, *p* < 0.001), while education was also independently predictive (OR = 0.574, 95% CI 0.464–0.699, *p* < 0.001) (Table 7). The variance inflation factor was 1.012, and adding IC1 significantly improved model fit (likelihood-ratio *χ*^2^ = 14.587, *df* = 1, *p* < 0.001).

**Table 6:** Cognition diagnostic performance: adjusted vs. raw CIST reference.

Metric	Adjusted CIST	Raw CIST <20	Raw − adjusted [95% CI]
Sensitivity	0.728	0.726	−0.001 [−0.033, 0.029]
Specificity	0.502	0.500	−0.002 [−0.023, 0.020]
AUC	0.615	0.613	−0.002 [−0.027, 0.024]
*κ*	0.215	0.212	−0.004 [−0.051, 0.045]

All difference CIs include zero, confirming low specificity is intrinsic, not artifactual.

**Table 7:** Independent association of ICOPE cognition screening with CIST-defined cognitive impairment.

Predictor	Adjusted OR [95% CI]	*p*
ICOPE cognition screen positive	2.219 [1.471, 3.371]	<0.001
Education level (per category)	0.574 [0.464, 0.699]	<0.001

Binary logistic regression (*N* = 464). VIF = 1.012. Adding IC1 improved fit: LR *χ*^2^ = 14.587, *p* < 0.001.

### 3.6 Locomotion domain: post-hoc sensitivity analysis for incorporation bias

Using a modified SPPB comprising only balance and gait speed subscores (range 0–8, cutoff ≤6), the diagnostic accuracy of IC6 decreased: sensitivity from 0.700 to 0.665, specificity from 0.836 to 0.676, AUC from 0.768 [0.729, 0.805] to 0.670 [0.631, 0.710], and Cohen’s *κ* from 0.499 to 0.340 (ΔAUC = −0.097 [95% CI − 0.134, −0.063]) (Supplementary Table S4). The AUC reduction quantifies the inflation attributable to the shared chair-stand component. Despite this reduction, the chair-rise test retained fair screening utility (AUC = 0.670) against the independent balance-gait reference.

### 3.7 Multivariate-adjusted urban–rural differences

Multivariate logistic regression adjusting for age, education, and sex (Table 5) revealed that among reference-positive participants, urban residence was associated with significantly higher detection sensitivity for cognition (adjusted OR = 4.821 [2.239, 11.154], *p* < 0.001) and vitality (OR = 4.096 [2.160, 8.031], *p* < 0.001), but significantly lower detection sensitivity for locomotion (OR = 0.211 [0.111, 0.387], *p* < 0.001). The psychological domain showed no significant urban–rural difference after adjustment (OR = 0.687 [0.226, 1.870], *p* = 0.480). All variance inflation factors were below 1.54, and results were materially unchanged when education was dichotomized.

### 3.8 Recruitment-source sensitivity analysis

To assess potential selection bias, we excluded the 157 participants recruited from the primary-care outpatient clinic (Table 8). In the community-only subsample (*n* = 307), locomotion AUC improved from 0.768 to 0.818 (Δ = +0.050 [0.021, 0.078]) and psychological AUC improved from 0.709 to 0.759 (Δ = +0.050 [−0.001, 0.104]). Cognition and vitality AUC showed modest, non-significant decreases (Δ = −0.025 and −0.031, respectively; both CIs crossing zero). Firth penalized logistic regression with BH-FDR correction showed that recruitment-source effects were domain- and metric-specific rather than uniform. Recruitment source was significantly associated with vitality sensitivity and specificity and with locomotion specificity, but not with sensitivity for cognition, psychological well-being, or locomotion. The paired comparisons of the augmented vitality strategies are presented in Table 9 and are discussed below. These findings indicate that recruitment setting may influence selected diagnostic metrics and should be considered when generalizing the results to unselected community populations (see Table 10).

**Table 8:** Sensitivity analysis excluding participants recruited from the primary-care outpatient site.

Domain	Metric	Full (*N* = 464)	Excl. primary-care (*n* = 307)	Δ [95% CI]
Cognition	Sn	0.728	0.654	−0.074 [−0.110, −0.034]
Cognition	Sp	0.502	0.525	+0.024 [−0.020, 0.068]
Cognition	AUC	0.615	0.590	−0.025 [−0.054, 0.004]
Vitality	Sn	0.590	0.438	−0.152 [−0.207, −0.094]
Vitality	Sp	0.700	0.790	+0.090 [0.055, 0.127]
Vitality	AUC	0.645	0.614	−0.031 [−0.065, 0.006]
Psychological	Sn	0.723	0.800	+0.077 [−0.017, 0.180]
Psychological	Sp	0.696	0.718	+0.022 [−0.006, 0.052]
Psychological	AUC	0.709	0.759	+0.050 [−0.001, 0.104]
Locomotion	Sn	0.700	0.769	+0.070 [0.032, 0.109]
Locomotion	Sp	0.836	0.866	+0.030 [−0.012, 0.073]
Locomotion	AUC	0.768	0.818	+0.050 [0.021, 0.078]
Any domain	AUC	0.671	0.731	+0.060 [0.032, 0.096]

Δ = excluded-site estimate minus full-sample estimate. Bootstrap 95% CI (*B* = 2000, seed = 42).

**Table 9:** Paired comparisons of augmented vitality strategies.

Comparison	Δ Sn [95% CI]	Δ Sp [95% CI]	Δ AUC [95% CI]	Paired *p* (Sn/Sp)
IC2 + BMI vs. IC2	+0.092 [0.054, 0.132]	−0.008 [−0.021, 0.000]	+0.042 [0.022, 0.063]	<0.001/0.500
IC2 + BMI + SNAQ vs. IC2	+0.230 [0.174, 0.285]	−0.097 [−0.135, −0.063]	+0.067 [0.031, 0.101]	<0.001/<0.001
IC2 + BMI + SNAQ vs. IC2 + BMI	+0.138 [0.095, 0.186]	−0.089 [−0.125, −0.057]	+0.025 [−0.005, 0.053]	<0.001/<0.001

McNemar-equivalent binomial tests. Bootstrap CIs (*B* = 2000, seed = 42).

**Table 10:** Adjusted recruitment-source associations with sensitivity and specificity.

Domain	Metric	Ref-stratum N	Adjusted OR [95% CI]	Nominal p	BH-FDR q
Cognition	Sensitivity	191	2.184 [0.632, 7.523]	0.214	0.290
Cognition	Specificity	273	0.542 [0.263, 1.100]	0.090	0.172
Vitality	Sensitivity	217	4.545 [1.875, 11.357]	<0.001	0.003
Vitality	Specificity	247	0.208 [0.080, 0.516]	<0.001	0.003
Psychological	Sensitivity	119	0.566 [0.119, 2.155]	0.417	0.417
Psychological	Specificity	345	0.705 [0.337, 1.460]	0.347	0.386
Locomotion	Sensitivity	293	1.694 [0.715, 4.093]	0.232	0.290
Locomotion	Specificity	171	0.080 [0.013, 0.355]	<0.001	0.003

Firth penalized logistic regression adjusted for residential area, age, low educational attainment, and sex. BH-FDR *q* < 0.05 indicates significance after multiple comparison correction.

## 4 Discussion

This study is the first Korean diagnostic accuracy study of the four assessable non-sensory domains of the WHO ICOPE Step 1 tool in community-dwelling older adults. Across these four domains, ICOPE Step 1 reached an overall sensitivity of 89.0% for detecting any IC decline, suggesting utility as an initial case-finding tool. Domain-specific performance varied considerably: the original SPPB-based locomotion estimate showed the highest apparent accuracy, whereas cognition showed the weakest specificity. These results align with the international validation literature but also reveal Korea-specific patterns relevant to ICOPE implementation in super-aged Asian societies.

Our overall sensitivity of 89.0% is somewhat lower than the 94%–96% reported from Hong Kong (6), Beijing (8), and the Chinese nursing-home cohort (9), but it exceeds the 63%–88% range reported in the Spanish VIMCI study (7). This discrepancy likely reflects population and recruitment differences: our sample had a relatively high mean age (78.4 years), a high proportion of participants with limited formal education (72.8%), and was recruited purposively from mixed community-based and service-linked sites rather than from a population registry. Our specificity of 45.1% falls within the 42%–58% range reported in community-based studies (6, 8) and confirms a recognized limitation of ICOPE Step 1: by design, it favors sensitivity at the expense of specificity.

The structural overlap between the IC6 chair-rise test and the SPPB chair-stand component introduced incorporation bias. Our modified SPPB analysis quantified this: the AUC decreased from 0.768 to 0.670 (Δ = −0.097 [95% CI − 0.134, −0.063]), and Cohen’s *κ* from 0.499 to 0.340 (Supplementary Table S4). This approximately 0.10-point AUC reduction indicates that the original locomotion accuracy represented an upper-bound estimate. Nevertheless, the modified AUC of 0.670 still indicates fair diagnostic performance. Accordingly, the primary SPPB-based locomotion estimates should be interpreted together with the modified SPPB analysis rather than as fully independent validation of the chair-rise item. Future studies should consider independent reference standards such as gait speed alone or the Timed Up and Go test.

The poor performance of the cognition domain (AUC = 0.615, *κ* = 0.215) is arguably our most clinically important finding. The three-word recall item produced a false-positive rate of nearly 50% among cognitively intact participants, and the problem was more pronounced in the low-education subgroup (specificity 45.7%) than in the higher-education subgroup (58.2%). This pattern echoes Leung et al. (6), who attributed a cognition sensitivity of 74.7% to participants with limited education, and Rodríguez-Laso et al. (23), who found that only 18% of even healthy older adults in the Toledo cohort successfully recalled three words. In our sample, 72.8% had elementary-school education or less, which amplifies this vulnerability. The CIST mitigates educational bias with age- and education-adjusted cutoffs, but the ICOPE Step 1 cognition item itself has no such calibration. These findings support education-stratified interpretation or confirmatory cognitive assessment for low-education Korean populations, rather than stand-alone classification based on the three-word recall item.

The 59.0% sensitivity of the vitality domain is in line with prior validation studies: Rojano i Luque et al. (7) reported 46%, Lu et al. (8) reported 51.3%, and Giudici et al. (24) reported 50%. The Step 1 vitality items (weight loss and appetite) appear insufficiently sensitive to the subclinical nutritional risk captured by the MNA-SF, which also accounts for BMI, mobility, neuropsychological problems, and acute disease. Lu et al. (8) showed that adding a BMI-based criterion (≤20 kg/m^2^) improved vitality sensitivity from 51% to 69%, and a similar adaptation may benefit the Korean implementation.

Because the standard ICOPE vitality items showed limited sensitivity, we performed a post-hoc analysis adding a BMI ≤ 20 kg/m^2^ criterion, in line with Lu et al. (8). The augmented screen increased sensitivity from 59.0% [95% CI 0.53, 0.66] to 68.2% [0.62, 0.74] (McNemar *p* < 0.001) while specificity remained essentially unchanged (70.0 to 69.2%) (Table 9). AUC and *κ* likewise improved (AUC 0.65 → 0.69; *κ* 0.29 → 0.37; full results in Table 11). These results indicate that incorporating BMI into the Korean ICOPE vitality pathway materially improves case-finding without sacrificing specificity.

**Table 11:** Diagnostic performance of standard and augmented vitality screening strategies (*N* = 464).

Strategy	Screen+, *n* (%)	Sn	Sp	PPV	NPV	AUC	*κ*
Standard IC2	202 (43.5%)	0.590	0.700	0.634	0.660	0.645	0.291
IC2 or BMI ≤ 20	224 (48.3%)	0.682	0.692	0.661	0.713	0.687	0.374
IC2 or BMI ≤ 20 or SNAQ <14	276 (59.5%)	0.820	0.603	0.645	0.793	0.712	0.417

MNA-SF ≤11. IC2 = standard ICOPE vitality items (weight loss + appetite).

The urban–rural divergence in screening performance is a new observation not reported in previous ICOPE validation studies. Urban residents showed markedly higher cognition sensitivity than rural residents (83.0% vs. 64.1%), likely because urban participants are more familiar with structured cognitive tasks. Rural residents, in contrast, showed substantially higher locomotion sensitivity (85.5% vs. 53.2%), perhaps because functionally limited older adults in agricultural communities, where daily life is physically demanding, are more likely to fail the chair-rise test. These setting-dependent differences suggest that a single, uniform national screening protocol may not perform consistently across Korea’s diverse residential contexts.

Multivariate logistic regression adjusting for age, education, and sex (Table 5) confirmed that the urban–rural locomotion screening disparity persists after demographic adjustment (adjusted OR = 0.211, *p* < 0.001), with urban residents having only 21% of the detection odds of rural residents among those with confirmed locomotion decline. For cognition and vitality, urban residence was associated with higher detection odds (cognition OR = 4.821; vitality OR = 4.096; both *p* < 0.001), potentially reflecting greater familiarity with structured assessment tasks in urban environments. The psychological domain showed no significant geographic difference after adjustment (*p* = 0.480). These findings support setting-sensitive interpretation rather than separate diagnostic rules for urban and rural residents, because residual confounding by severity, task familiarity, occupational history, and unmeasured contextual factors may remain.

The self-reported sensory items revealed a large discrepancy between perceived current difficulty and recalled medical diagnosis: 45.0% reported hearing difficulty versus 6.2% reporting a prior diagnosis of hearing loss, and 64.7% reported vision difficulty versus 12.9% reporting a prior diagnosis of cataract/glaucoma. Because both variables were self-reported and no audiometric or ophthalmologic reference standard was available, this discrepancy should not be interpreted as diagnostic validation or as direct evidence of underdiagnosis. Rather, it highlights the need to pair ICOPE sensory questions with objective hearing and vision screening in future Korean validation studies.

### 4.1 Clinical implications for implementation

Our findings suggest several implementation refinements for Korean community settings, but these should be interpreted as proposals requiring prospective validation rather than replacement algorithms for the WHO ICOPE Step 1 protocol. First, for cognition, an education-stratified pathway may be needed: older adults with no formal education could proceed directly to CIST or another brief secondary cognitive assessment, whereas those with elementary education who screen positive on the three-word recall item should be considered pending CIST confirmation rather than definitively classified as cognitively impaired. Second, for locomotion, the chair-rise item should not be used as a sole rule-out test; a positive result may help prioritize Step 2 assessment, but negative results should be interpreted alongside gait, balance, fall history, and functional complaints. Third, for vitality, adding BMI ≤ 20 kg/m^2^ to the standard ICOPE vitality items improved sensitivity without meaningful specificity loss and may be feasible where height and weight are routinely measured. Fourth, sensory questions should be retained as initial flags but paired with objective hearing and vision screening where feasible. These implementation proposals are being examined prospectively in the DREAMS Year 2 study.

### 4.2 Strengths and limitations

This study has several strengths. It is the first Korean diagnostic accuracy study of four assessable non-sensory ICOPE Step 1 domains in community-dwelling older adults, addressing a gap explicitly noted in the Korean geriatric policy literature (13). With 464 participants, the sample is larger than most previous validation cohorts (6–8) and spans both urban and rural community settings. The use of the CIST with age- and education-adjusted cutoffs also provides a more contextually appropriate cognition reference than the MMSE used in earlier studies. Post-hoc sensitivity analyses addressed several reviewer-identified methodological concerns, including locomotion incorporation bias, education effects on cognition specificity, demographic confounding of urban–rural comparisons, prevalence-dependent predictive values, and recruitment-source selection bias.

This study also has several limitations. First, the cross-sectional design precludes assessment of predictive validity, temporal stability, or downstream clinical outcomes. Second, because objective audiometric and ophthalmologic assessments were not performed, hearing and vision were reported descriptively only; the present study therefore represents a partial four-domain validation rather than validation of the complete ICOPE Step 1 framework. Third, complete assessor blinding was not feasible because the same trained research team administered the index and reference assessments during the same session. Fourth, purposive sampling from community-based and service-linked recruitment sites may limit generalizability to unselected older adults living independently in the community; therefore, observed PPV and NPV should be interpreted as sample-specific, and the prevalence-adjusted estimates in Table 12 should be interpreted as scenario-based estimates for public-health planning. Fifth, recruitment-source effects were domain- and metric-specific rather than absent; significant source effects were observed for vitality and locomotion specificity. Sixth, the primary SPPB reference standard for locomotion included a chair-stand component overlapping with the ICOPE chair-rise item; the modified SPPB analysis quantified the resulting incorporation bias. Seventh, diagnosed hearing and vision conditions were based on self-reported medical history rather than objective clinical confirmation. Finally, data came from a single urban–rural mixed municipality, and several subgroup and post-hoc analyses should be considered exploratory pending external validation.

**Table 12:** Prevalence-adjusted PPV and NPV across assumed domain prevalences.

Domain	Sn	Sp	*p* = 10% PPV/NPV	*p* = 20% PPV/NPV	*p* = 30% PPV/NPV	*p* = 40% PPV/NPV	*p* = 50% PPV/NPV
Cognition	0.728	0.502	14.0/94.3	26.8/88.1	38.5/81.1	49.3/73.4	59.4/64.8
Vitality	0.590	0.700	17.9/93.9	33.0/87.2	45.8/79.9	56.8/71.9	66.3/63.1
Psychological	0.723	0.696	20.9/95.8	37.3/90.9	50.4/85.4	61.3/79.0	70.4/71.5
Locomotion	0.700	0.836	32.2/96.2	51.6/91.8	64.7/86.7	74.0/80.7	81.0/73.6

Each cell reports PPV/NPV (%). Computed using Bayes’ theorem with observed Sn and Sp held constant.

## 5 Conclusion

In this cross-sectional diagnostic accuracy study, the four assessable non-sensory domains of ICOPE Step 1 showed acceptable overall sensitivity but heterogeneous domain-specific accuracy in Korean community-dwelling older adults. The cognition item showed limited specificity, particularly among older adults with limited formal education, supporting the need for education-stratified cognitive confirmation. The vitality domain may benefit from BMI-augmented screening. The original locomotion estimates were partly inflated by incorporation bias from the SPPB chair-stand component, although the modified SPPB analysis suggested fair independent screening utility. These findings support cautious, context-sensitive implementation of ICOPE Step 1 in Korean community settings and highlight the need for future validation including objective hearing and vision reference standards.

## Data Availability

The datasets presented in this article are not readily available because they contain participant-level data subject to ethical and privacy restrictions. Requests to access the datasets should be directed to the corresponding author, MP (doogroop@gnu.ac.kr), and will be considered subject to institutional approval.
