# Correction: Diagnostic accuracy of the WHO ICOPE step 1 screening tool in Korean community-dwelling older adults: a cross-sectional validation study

**DOI:** 10.3389/fpubh.2026.1970851

**Published:** 2026-09-08

**Authors:** HeeKyung Chang, MinJi Park, JuHee Seo, YoungJoo Do

**Affiliations:** College of Nursing, Gyeongsang National University, Jinju, Republic of Korea

**Keywords:** community-dwelling, diagnostic accuracy, healthy aging, ICOPE, intrinsic capacity, Korea, older adults, screening

In the published article, there was an error in the reported data-collection period. The data-collection period was incorrectly stated as “between March and December 2024.” The correct data-collection period is “between October 2025 and January 2026.” This correction concerns only the reported data-collection period and does not affect the participant data, measurements, statistical analyses, results, or conclusions of the study.

A correction has been made to the section **2 Methods**, **2.1 Study design and setting**, Paragraph 1:

“This cross-sectional diagnostic accuracy study was conducted in Jinju, a city in Gyeongnam Province, South Korea. Jinju is an urban–rural mixed municipality (DoNong Bokhap) that includes both urban districts (Eup/Dong) and rural districts (Myeon), and thus provides a pragmatic urban–rural mixed setting for evaluating ICOPE screening across common Korean community service environments. Data were collected between October 2025 and January 2026 as part of the Digital Rural–urban Ecosystem for Active Management and Successful Aging (DREAMS) project, a multi-year community-based program on healthy aging funded by the National Research Foundation of Korea.”

The original version of this article has been updated.

