# Supplementary material for: Diagnostic accuracy of the WHO ICOPE step 1 screening tool in Korean community-dwelling older adults: a cross-sectional validation study
Source: Front Public Health. 2026 Jul 30;14:1897282. doi: 10.3389/fpubh.2026.1897282 (PMC13471507; doi:10.3389/fpubh.2026.1897282)
Supplement: Supplementary file 1 [file Data_sheet_1.docx]

**Supplementary Material**

*Diagnostic accuracy of the WHO ICOPE Step 1 screening tool in Korean community-dwelling older adults: a cross-sectional validation study*

Manuscript ID: 1897282 | Frontiers in Public Health

**Contents:**

Supplementary Table S1. 2×2 contingency tables for each ICOPE Step 1 domain

Supplementary Table S2. Sensory domain cross-tabulation

Supplementary Table S3. Distribution of number of domains with IC decline

Supplementary Table S4. Modified SPPB sensitivity analysis (incorporation bias)

Supplementary Table S5. STARD 2015 checklist

Supplementary Table S6. Participant characteristics by recruitment-site category

**Supplementary Table S1. 2×2 contingency tables for ICOPE Step 1 domains (N = 464)**

**Cognition**

|  | **Reference +** | **Reference −** | **Total** |
| --- | --- | --- | --- |
| Screen + | 139 | 136 | 275 |
| Screen − | 52 | 137 | 189 |
| Total | 191 | 273 | 464 |

**Vitality**

|  | **Reference +** | **Reference −** | **Total** |
| --- | --- | --- | --- |
| Screen + | 128 | 74 | 202 |
| Screen − | 89 | 173 | 262 |
| Total | 217 | 247 | 464 |

**Psychological**

|  | **Reference +** | **Reference −** | **Total** |
| --- | --- | --- | --- |
| Screen + | 86 | 105 | 191 |
| Screen − | 33 | 240 | 273 |
| Total | 119 | 345 | 464 |

**Locomotion**

|  | **Reference +** | **Reference −** | **Total** |
| --- | --- | --- | --- |
| Screen + | 205 | 28 | 233 |
| Screen − | 88 | 143 | 231 |
| Total | 293 | 171 | 464 |

**Any domain (≥1)**

|  | **Reference +** | **Reference −** | **Total** |
| --- | --- | --- | --- |
| Screen + | 340 | 45 | 385 |
| Screen − | 42 | 37 | 79 |
| Total | 382 | 82 | 464 |

**Supplementary Table S2. Sensory domain: ICOPE Step 1 self-report vs. self-reported diagnoses**

**Hearing**

|  | **Diagnosed +** | **Diagnosed −** | **Total** |
| --- | --- | --- | --- |
| Self-report + | 26 | 183 | 209 |
| Self-report − | 3 | 252 | 255 |
| Total | 29 | 435 | 464 |

**Vision**

|  | **Diagnosed +** | **Diagnosed −** | **Total** |
| --- | --- | --- | --- |
| Self-report + | 52 | 248 | 300 |
| Self-report − | 8 | 156 | 164 |
| Total | 60 | 404 | 464 |

*Diagnosed conditions based on self-reported medical history from chronic disease checklist, not objective clinical assessment.*

**Supplementary Table S3. Distribution of number of IC domains with decline**

| **Number of domains** | **Step 1 screening, n (%)** | **Reference standard, n (%)** |
| --- | --- | --- |
| 0 | 79 (17.0%) | 82 (17.7%) |
| 1 | 109 (23.5%) | 120 (25.9%) |
| 2 | 145 (31.3%) | 138 (29.7%) |
| 3 | 96 (20.7%) | 87 (18.8%) |
| 4 | 35 (7.5%) | 37 (8.0%) |

*Based on four assessable domains (cognition, vitality, psychological, locomotion). Sensory domains excluded.*

**Supplementary Table S4. Locomotion diagnostic performance using original and modified SPPB reference standards**

| **Metric** | **Original SPPB ≤9** | **Modified SPPB ≤6** | **Modified − original [95% CI]** |
| --- | --- | --- | --- |
| Reference positive, n | 293 | 242 | −51 |
| Sensitivity | .700 | .665 | −.034 [−.062, −.007] |
| Specificity | .836 | .676 | −.161 [−.215, −.114] |
| PPV | .880 | .691 | −.189 [−.245, −.141] |
| NPV | .619 | .649 | +.030 [−.008, .066] |
| AUC | .768 | .670 | −.097 [−.134, −.063] |
| κ | .499 | .340 | −.159 [−.225, −.095] |

*Modified SPPB = balance score + 4-m gait score (0–8), excluding the chair-stand component. Cutoff ≤6. 2×2 counts (TP/FP/FN/TN): original 205/28/88/143; modified 161/72/81/150. The ΔAUC of −0.097 quantifies the incorporation bias from the shared chair-stand component.*

**Supplementary Table S5. STARD 2015 Checklist**

*[See attached STARD 2015 checklist document]*

**Supplementary Table S6. Participant characteristics by recruitment-site category (N = 464)**

| **Variable** | **Total (N=464)** | **Senior welfare center (n=81)** | **Primary-care outpatient (n=157)** | **Day-care center (n=122)** | **Community hall (n=104)** | ***p*** |
| --- | --- | --- | --- | --- | --- | --- |
| Age, mean (SD) | 78.4 (7.6) | 78.4 (4.9) | 76.5 (6.7) | 84.1 (7.1) | 74.5 (7.3) | <.001 |
| Female, n (%) | 315 (67.9) | 29 (35.8) | 126 (80.3) | 92 (75.4) | 68 (65.4) | <.001 |
| Edu ≤elementary, n (%) | 338 (72.8) | 23 (28.4) | 141 (89.8) | 104 (85.2) | 70 (67.3) | <.001 |
| Urban, n (%) | 268 (57.8) | 81 (100.0) | 157 (100.0) | 30 (24.6) | 0 (0.0) | <.001 |
| CIST, mean (SD) | 20.6 (7.2) | 23.5 (4.9) | 20.6 (8.2) | 17.4 (6.4) | 21.9 (6.6) | <.001 |
| SPPB, mean (SD) | 8.4 (2.5) | 10.1 (1.4) | 8.1 (2.7) | 7.3 (2.5) | 8.7 (1.8) | <.001 |
| FPQ ≥3, n (%) | 224 (48.3) | 17 (21.0) | 86 (54.8) | 84 (68.9) | 37 (35.6) | <.001 |
| IC decline ≥1, n (%) | 382 (82.3) | 46 (56.8) | 138 (87.9) | 114 (93.4) | 84 (80.8) | <.001 |

*Welch ANOVA for continuous variables; Pearson chi-square for categorical variables. Categories: 2 senior welfare centers, 1 primary-care outpatient clinic, 3 day-care centers, 5 community halls/village centers.*
