# Supplementary material for: Diagnostic accuracy of the WHO ICOPE step 1 screening tool in Korean community-dwelling older adults: a cross-sectional validation study
Source: Front Public Health. 2026 Jul 30;14:1897282. doi: 10.3389/fpubh.2026.1897282 (PMC13471507; doi:10.3389/fpubh.2026.1897282)
Supplement: Supplementary file 2 [file Data_sheet_2.docx]

**Supplementary Table S5. STARD 2015 Checklist**

*Manuscript: Diagnostic accuracy of the WHO ICOPE Step 1 screening tool in Korean community-dwelling older adults (MS#1897282)*

| **Section** | **#** | **STARD 2015 Item** | **Location in Manuscript** |
| --- | --- | --- | --- |
| **TITLE/ABSTRACT** | | | |
|  | **1** | Identification as a study of diagnostic accuracy using at least one measure of accuracy (such as sensitivity, specificity, predictive values, or AUC) | Title: "Diagnostic accuracy of the WHO ICOPE Step 1 screening tool..." Abstract: sensitivity, specificity, PPV, NPV, AUC, and Cohen's kappa reported |
| **ABSTRACT** | | | |
|  | **2** | Structured summary of study design, methods, results, and conclusions | Abstract provides structured summary with Background, Methods, Results, and Conclusion sections |
| **INTRODUCTION** | | | |
|  | **3** | Scientific and clinical background, including the intended use and clinical role of the index test | Section 1 (Introduction): ICOPE Step 1 as community screening tool for IC decline; intended use in Korean primary-care and community settings |
|  | **4** | Study objectives and hypotheses | Section 1, final paragraph: three specific aims stated (domain-level accuracy, overall screening accuracy, subgroup variation) |
| **METHODS** | | | |
|  | **5** | Whether data collection was planned before the index test and reference standard were performed (prospective study) or after (retrospective study) | Section 2.1: Cross-sectional diagnostic accuracy study; data collected prospectively March–December 2024 as part of the DREAMS project |
|  | **6** | Eligibility criteria | Section 2.2: Aged ≥65, community-dwelling in Jinju, able to consent. Exclusions: acute illness, severe cognitive impairment, hospitalization |
|  | **7** | On what basis potentially eligible participants were identified (such as symptoms, results from previous tests, inclusion in registry) | Section 2.2: Recruited from 4 categories of community partner institutions (senior welfare centers, primary-care outpatient clinic, day-care centers, community halls) |
|  | **8a** | Index test, in sufficient detail to allow replication | Section 2.4: WHO ICOPE Step 1 tool, with six conditions across five intrinsic-capacity domains, described with specific items. |
|  | **8b** | Reference standard, in sufficient detail to allow replication | Section 2.5: CIST (age/education-adjusted cutoffs specified), MNA-SF ≤11, GDS-15 ≥8, SPPB ≤9. Cutoff values and scoring details provided |
|  | **9** | Definition of and rationale for test positivity cut-offs or result categories of the index test, distinguishing pre-specified from exploratory | Section 2.4: Each domain coded as screen-positive/negative per ICOPE handbook protocol (pre-specified). Post-hoc BMI augmentation and modified SPPB described as exploratory |
|  | **10a** | Whether clinical information and reference standard results were available to the performers/readers of the index test | Not explicitly stated. However, ICOPE Step 1 was administered as first assessment; reference standards administered subsequently by the same research team |
|  | **10b** | Whether clinical information and index test results were available to the assessors of the reference standard | Not explicitly stated. Both tests administered in same session. Acknowledged as limitation: blinding was not feasible in this community screening context |
|  | **11** | Rationale for choosing the reference standard (if alternatives exist) | Section 2.5: CIST chosen over MMSE for Korean-specific age/education-adjusted cutoffs; MNA-SF, GDS-15, SPPB selected as validated Korean-language instruments |
|  | **12a** | Definition of and rationale for test positivity cut-offs or result categories of the reference standard, distinguishing pre-specified from exploratory | Section 2.5: All cutoffs pre-specified with citations (CIST: age/education-adjusted from manual; MNA-SF ≤11; GDS-15 ≥8 per Korean community validation; SPPB ≤9) |
|  | **12b** | If the reference standard was not applied to all participants, describe the rationale for selective application | All reference standards applied to all 464 participants; sensory domains excluded due to absence of objective audiometric/ophthalmologic assessment (Section 2.5) |
|  | **13a** | Whether participants were enrolled consecutively, randomly, or by convenience | Section 2.2: Purposive sampling to ensure balanced urban/rural representation; acknowledged as limitation in Section 4.2 |
|  | **13b** | If participants were not recruited at the same time, describe the time interval during which participants were recruited | Section 2.1: March–December 2024 |
|  | **14** | Sample size: how sample size was determined, and whether it was before the study began | Not explicitly stated as a formal power calculation. Sample of 464 described; larger than most prior ICOPE validation studies (Discussion) |
| **RESULTS** | | | |
|  | **15** | Time intervals and any clinical interventions between the index test and reference standard | Both administered in same assessment session; no time interval or intervention between tests |
|  | **16** | The number of eligible participants who did or did not undergo the index test and/or reference standard; describe reasons for non-participation | Section 3.1: All 464 enrolled participants completed both index test and reference standards; no missing data reported. Flow diagram not provided (all participants completed all tests) |
|  | **17** | Distribution of severity of disease in those with the target condition and demographics and clinical features of the study population | Table 1: Full demographic characteristics. Table 2: Prevalence of IC decline by domain. Supplementary Table S6: Characteristics by recruitment site |
|  | **18** | Cross-tabulation of the index test results (or their distribution) by the results of the reference standard | Table 3: 2×2 contingency table metrics. Supplementary Table S1: Full 2×2 tables for each domain |
|  | **19** | Estimates of diagnostic accuracy and their precision (such as 95% confidence intervals) | Table 3: Sn, Sp, PPV, NPV, AUC, κ with 95% bootstrap CIs for all domains |
|  | **20** | Any adverse events from performing the index test or the reference standard | No adverse events occurred (not explicitly reported; community-based non-invasive assessments) |
|  | **21** | Table of any subgroup analyses or exploratory analyses | Table 4: subgroup analyses by education, sex, and residential area. Tables 5–12: exploratory and sensitivity analyses, including adjusted urban–rural differences, cognition calibration, recruitment-source sensitivity, augmented vitality strategies, and prevalence-adjusted predictive values. Supplementary Table S4: modified SPPB analysis. |
| **DISCUSSION** | | | |
|  | **22** | Study limitations, including sources of potential bias, statistical uncertainty, and generalizability | Section 4.2: Limitations discussed include the cross-sectional design, sensory-domain exclusion, incomplete assessor blinding, purposive sampling, recruitment-source effects, incorporation bias, self-reported sensory diagnoses, and the single-municipality setting. |
|  | **23** | Implications for practice, including the intended use and clinical role of the index test | Section 4.1: Implementation recommendations address education-stratified cognitive confirmation, cautious interpretation of locomotion screening, BMI-augmented vitality screening, and objective confirmation of sensory decline. Section 5 summarizes the clinical implications. |
| **OTHER** | | | |
|  | **24** | Registration number and name of registry | Not applicable (diagnostic accuracy study; not a clinical trial). IRB: GIRB-G25-NW-0112 |
|  | **25** | Where the full study protocol can be accessed | Not separately published. Study conducted as part of DREAMS project (NRF RS-2025-00558940). Data available from corresponding author on reasonable request |
|  | **26** | Sources of funding and other support; role of funders | Funding section: National Research Foundation of Korea (NRF), Ministry of Science and ICT (No. RS-2025-00558940). Conflict of Interest: none declared |

*STARD = Standards for Reporting Diagnostic Accuracy Studies. Checklist based on Bossuyt PM, Reitsma JB, Bruns DE, et al. STARD 2015: an updated list of essential items for reporting diagnostic accuracy studies. BMJ. 2015;351:h5527.*
